# When Platelets Fail: A Case of Active Gastrointestinal Bleeding in Immune Thrombocytopenia

**DOI:** 10.7759/cureus.85927

**Published:** 2025-06-13

**Authors:** Tahreem Malik, Marion Capahi

**Affiliations:** 1 Department of Internal Medicine, Health Corporation of America Healthcare/University of South Florida Morsani College of Medicine Graduate Medical Education: Health Corporation of America Citrus, Inverness, USA

**Keywords:** acute gi bleed, blood platelets, heme complications, immune-hematology, immune thrombocytopenia (itp)

## Abstract

Immune thrombocytopenic purpura (ITP) is an acquired autoimmune disorder characterized by the destruction of platelets due to autoantibodies, leading to an increased risk of bleeding. This case report describes a 77-year-old female who presented with acute gastrointestinal bleeding, severe thrombocytopenia, and mucocutaneous purpura. Initial laboratory evaluation revealed a critically low platelet count of <5 ×10³/μL, prompting urgent management with platelet transfusions, intravenous corticosteroids, and intravenous immunoglobulin (IVIG). Despite initial treatment, her thrombocytopenia persisted, necessitating the administration of the thrombopoietin receptor agonist romiplostim, which led to sustained platelet recovery. Further investigations revealed a recent history of an upper respiratory infection treated with antibiotics and newly diagnosed hepatitis C, highlighting potential infectious and medication-related triggers. This case underscores the complexity of ITP diagnosis and management, emphasizing the need for individualized treatment strategies, particularly in cases of severe bleeding. It also reinforces the importance of early recognition and timely intervention to prevent life-threatening complications.

## Introduction

Immune thrombocytopenic purpura (ITP) is an acquired autoimmune disorder characterized by isolated thrombocytopenia, defined as a platelet count below 100 ×10³/μL, in the absence of other identifiable causes. It arises due to immune-mediated destruction of platelets and impaired platelet production, driven by autoantibodies targeting platelet surface glycoproteins. While the pathogenesis of ITP is not completely understood, it is thought to have a complex interplay between genetics and environmental factors [1]. Thrombocytopenia, the key laboratory abnormality in ITP, can range in severity and clinical impact, from asymptomatic laboratory findings to spontaneous mucocutaneous bleeding or life-threatening hemorrhage.

Thrombocytopenia can result from a wide variety of conditions and must be carefully evaluated to determine the underlying cause. It may be due to decreased platelet production (as seen in bone marrow suppression from infections, malignancies, or chemotherapy), increased platelet destruction (as in ITP, thrombotic microangiopathies, or disseminated intravascular coagulation), or sequestration (commonly from splenomegaly). Drug-induced thrombocytopenia, viral infections such as HIV or hepatitis C, and autoimmune disorders like systemic lupus erythematosus can also mimic or trigger secondary forms of ITP. Therefore, distinguishing primary ITP from other causes of thrombocytopenia is essential for appropriate management. Although the pathophysiology of ITP is not completely understood, various treatment strategies have been developed to manage the disease and improve platelet counts [2].

This case report describes a 77-year-old female who presented with acute gastrointestinal bleeding, severe thrombocytopenia, and mucocutaneous purpura. Initial laboratory evaluation revealed a critically low platelet count of <5 ×10³/μL, prompting urgent management with platelet transfusions, intravenous corticosteroids, and intravenous immunoglobulin (IVIG). Despite initial treatment, her thrombocytopenia persisted, necessitating the administration of the thrombopoietin receptor agonist romiplostim, which led to sustained platelet recovery. Further investigations revealed a recent history of an upper respiratory infection treated with antibiotics and newly diagnosed hepatitis C, highlighting potential infectious and medication-related triggers.

This case underscores the complexity of diagnosing and managing ITP, especially in elderly patients presenting with severe bleeding. It reinforces the importance of a comprehensive evaluation to rule out secondary causes of thrombocytopenia and the need for individualized treatment strategies. Early recognition and timely intervention remain critical to preventing life-threatening complications and optimizing patient outcomes.

## Case presentation

A 77-year-old Caucasian female presented to the emergency department (ED) with a two-day history of acute rectal bleeding. Her past medical history was significant for hypertension and hypothyroidism. The patient reported that symptoms began three days prior to admission with an episode of hematemesis while watching television. Shortly thereafter, she experienced lightheadedness and dizziness upon standing. The following morning, she had another episode of dark vomitus followed by an episode of bright red emesis. Over the next 24 hours, she developed dark, progressively looser stools which transitioned to melena. These symptoms prompted her ED visit.

On further evaluation, the patient reported a recent upper respiratory tract infection approximately three weeks prior, for which she had completed a course of azithromycin and cefuroxime. She denied any lingering respiratory symptoms. She admitted to frequent use of over-the-counter medications, including BC Powder (containing 825 mg aspirin and caffeine), which she reported using multiple times weekly for joint pain. She also endorsed the use of several vitamins and dietary supplements.

**Figure 1:**
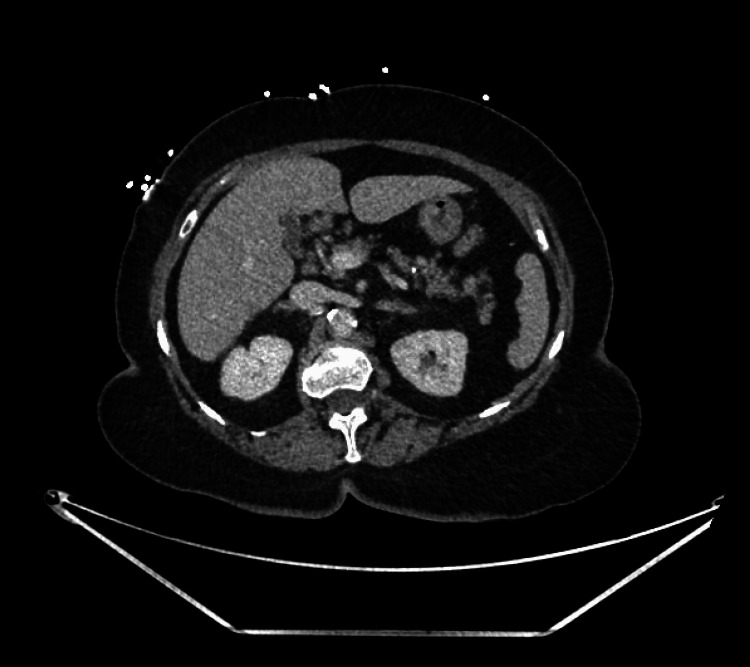
CT abdomen/pelvis

On initial evaluation, vital signs were notable for mild tachycardia, but she was normotensive. Physical examination revealed multiple purpuric lesions on the bilateral lower extremities, which the patient stated had been present for approximately one week. Digital rectal exam confirmed melena. Laboratory investigations demonstrated significant cytopenias (Table 1), with a hemoglobin of 6.3 g/dL, hematocrit of 17.6%, platelet count of 5 ×10⁹/L, white blood cell count of 15.8 ×10⁹/L, prothrombin time (PT) of 12.9 seconds, and INR of 1.1. Renal and liver function tests were within normal limits. CT Imaging of the abdomen and pelvis was unremarkable and did not reveal any sources of gastrointestinal bleeding (Figure 1).

**Table 1:** Blood count progression Hb: Hemoglobin, Hct: Hematocrit.

	Units	Reference Range	Day 0	Day 1	Day 1.5	Day 2	Day 2.5	Day 3	Day 4	Day 5	Day 6	Day 7	Day 8	1-Month follow-up	8 Weeks post-hospitalization	6-Month follow-up
WBC	(x10^3^/ul)	4-10	15.8	12.8		14.4		15.2	13.2	9.1	8	7.1	7.6	4.5	10.2	4.4
Hb	(g/dl)	12-15.5	5.2	6.6	7.3	8.1	8.2	9.4	9.2	8.8	9.3	9.6	10.7	11.3	10.5	12.2
Hct	(%)	36-44	14.3	17.6	20	22.7	23.4	26.9	25.9	25.6	27.6	28.3	31.3	33	30.8	34
Platelets	(x10^3^)	150-450	<5	44	22	34	6	26	28	41	36	17	31	313	57	176

Initial management included transfusion with 1 unit packed red blood cells (pRBCs), 1 unit fresh frozen plasma (FFP), and 1 unit platelet concentrate, as the underlying cause of her bleeding was unknown. Given the severe thrombocytopenia and concern for an underlying immune-mediated process, the patient received intravenous corticosteroids. A peripheral blood smear was performed and no evidence of schistocytes, blasts, or dysplastic features was found. A bone marrow biopsy was subsequently performed and demonstrated adequate trilineage hematopoiesis without evidence of marrow infiltration, myelodysplasia, or aplastic processes, effectively ruling out hematologic malignancy or marrow failure as the cause of her thrombocytopenia.

Due to ongoing hematemesis and melena, an upper endoscopic evaluation was considered. However, this was deferred due to the critically low platelet count and the associated risk of procedural bleeding. She was managed conservatively with intravenous proton pump inhibitor (PPI) therapy, intravenous fluids, and transfusion support as clinically indicated. Despite continued corticosteroid therapy, her platelet counts remained critically low, and gastrointestinal bleeding persisted. Intravenous immunoglobulin (IVIG) was initiated.

Given the presentation and lack of secondary causes on initial workup, immune thrombocytopenic purpura (ITP) was strongly suspected. A broader infectious disease workup was conducted. Serologies for HIV and hepatitis B were negative; however, the patient tested positive for hepatitis C virus (HCV), a diagnosis previously unknown to her. Epstein-Barr virus (EBV) immunoglobulin (Ig)M was also positive, although IgG and PCR were negative, suggesting a remote or false-positive result rather than acute infection.

Following IVIG therapy, the patient's platelet count improved to a level deemed safe for discharge. She was discharged with close outpatient hematology and gastrointestinal (GI) follow-up. Over the subsequent weeks, she experienced persistent thrombocytopenia despite an initial response. She was initiated on subcutaneous romiplostim, a thrombopoietin receptor agonist, with subsequent improvement in platelet counts to 313 ×10⁹/L within one month. However, eight weeks after initial presentation, she experienced a relapse in thrombocytopenia, which was managed successfully with another pulse of IVIG. She remains on maintenance romiplostim injections every two months, along with continued treatment of her HCV with stabilization of platelet counts (Table 1).

## Discussion

This case underscores the diagnostic and therapeutic complexities associated with immune thrombocytopenic purpura (ITP) in elderly patients, particularly when complicated by gastrointestinal bleeding and secondary etiologies such as hepatitis C virus (HCV) infection. ITP is increasingly recognized in older adults, with approximately half of newly diagnosed cases occurring in patients over the age of 60 [3]. In the elderly, ITP may present as primary or be secondary to conditions such as viral infections, autoimmune disorders, or medications. Hepatitis C virus (HCV) has been implicated in immune-mediated thrombocytopenia through mechanisms including molecular mimicry, immune complex formation, and impaired platelet production due to direct viral suppression of megakaryocytes [4].

Distinguishing ITP from other causes of thrombocytopenia, such as drug-induced thrombocytopenia, myelodysplastic syndromes, lymphoproliferative disorders, or thrombotic microangiopathies, is crucial in older adults [5]. This patient’s peripheral blood smear showed no schistocytes or blasts, and bone marrow biopsy demonstrated normal trilineage hematopoiesis, supporting a diagnosis of ITP. The recent upper respiratory infection and newly diagnosed HCV further suggested a secondary form of ITP. Although platelet antibody testing can be performed in suspected cases of ITP, it is not part of the recommended guideline and thus was omitted in this patient.

The management of ITP is centered on reducing bleeding risk by increasing platelet counts to a hemostatic level, typically above 30-50 ×10⁹/L. First-line treatment consists of corticosteroids, which act by suppressing autoantibody-mediated platelet destruction [2]. Although effective, long-term corticosteroid use is limited in elderly patients due to adverse effects, including hyperglycemia, osteoporosis, and increased susceptibility to infections. IVIG is frequently used in combination with steroids, especially in the setting of active bleeding or when a rapid platelet response is desired, as it typically elevates platelet counts within 24 to 48 hours [2]. In this case, the patient was treated with both corticosteroids and IVIG, but showed inadequate platelet recovery, necessitating second-line therapy. Thrombopoietin receptor agonists (TPO-RAs), such as romiplostim, have emerged as effective and well-tolerated options in chronic or refractory ITP, including in the elderly. Romiplostim was initiated in our patient with favorable and sustained improvement in platelet counts. Other second-line options include rituximab, a monoclonal antibody targeting CD20 on B cells, though its use may be limited in older adults due to delayed onset of action and heightened infection risk [2]. Splenectomy, once the standard second-line treatment, is now less commonly performed, particularly in elderly patients, due to perioperative risks and the potential for long-term complications such as overwhelming postsplenectomy infection. Nevertheless, it remains a consideration in select refractory cases. Supportive care measures, including platelet transfusions for life-threatening hemorrhage, proton pump inhibitors to reduce gastrointestinal bleeding, and discontinuation of antiplatelet medications, are also critical components of management. In this case, avoidance of further aspirin-containing products and initiation of romiplostim proved instrumental in achieving clinical stability.

TPO-RAs have demonstrated efficacy in chronic ITP, including in elderly populations [6]. These agents stimulate platelet production and can help maintain long-term hemostasis. However, caution is warranted due to an associated risk of thromboembolic events, which is particularly relevant in older patients with comorbidities. In our patient, romiplostim led to a significant and sustained increase in platelet count, allowing long-term outpatient management [6]. Endoscopic evaluation, while typically indicated in cases of upper gastrointestinal bleeding, was deferred in this patient due to profound thrombocytopenia. Guidelines suggest that invasive procedures should be avoided or delayed until platelet counts are at a safer threshold (generally ≥50 ×10⁹/L) unless emergent intervention is needed [7].

## Conclusions

This case highlights the diagnostic and therapeutic challenges of managing severe thrombocytopenia with concurrent gastrointestinal bleeding in an elderly patient. While ITP is a diagnosis of exclusion, it should be strongly considered in patients presenting with isolated thrombocytopenia after ruling out marrow failure, thrombotic microangiopathies, disseminated intravascular coagulation, and drug-induced causes. In this patient, recent upper respiratory infection, regular use of aspirin-containing over-the-counter medications, and newly diagnosed hepatitis C infection raised suspicion for secondary ITP. HCV is a well-recognized trigger for immune-mediated cytopenias, likely through chronic immune activation.

The patient’s severe bleeding and critically low platelet count precluded endoscopic evaluation, necessitating a non-invasive management approach focused on proton pump inhibitors, fluid resuscitation, blood product transfusions, and immunomodulatory therapies. Lack of response to corticosteroids alone prompted escalation to IVIG, followed by initiation of romiplostim, which achieved a durable platelet response. This case underscores the importance of a stepwise, individualized treatment strategy in elderly patients with refractory ITP, as well as the role of multidisciplinary collaboration. Careful monitoring and long-term follow-up are essential, as relapses may occur even after initial stabilization.
